# Analysis of antibiotics selection in patients undergoing appendectomy in a Chinese tertiary care hospital

**DOI:** 10.1186/s40064-016-3461-1

**Published:** 2016-10-21

**Authors:** Shengyong Xu, Xuezhong Yu, Yi Li, Donglei Shi, Jingya Huang, Qian Gao, Ting Zhang, Shigong Guo

**Affiliations:** 1 Department of Emergency Medicine, Peking Union Medical College Hospital, Peking Union Medical College, Chinese Academy of Medical Sciences, Beijing, 100730 China; 2 Department of Emergency Medicine, Beijing Shijitan Hospital, Capital Medical University, Beijing, China; 3 Saint Louis University School of Medicine, Class of 2014, 1042 S. Grand Blvd, Saint Louis, MO 63104 USA; 4 Trauma and Orthopaedic Surgery, Charing Cross Hospital, London, UK

**Keywords:** Appendicitis, Antibiotics, Appendectomy

## Abstract

**Background:**

To investigate the status of antibiotics use in acute appendicitis patients who undergo appendectomy in a Chinese tertiary care hospital.

**Methods:**

A retrospective analysis of 93 patients who underwent appendectomy from June 1, 2011 to May 30, 2012 and had recorded use of intravenous antibiotics. We defined simple appendicitis and suppurative appendicitis as mild appendicitis. Gangrenous appendicitis and perforated appendicitis were classified as advanced appendicitis. The occurrence of advanced appendicitis, postoperative complications and length of hospital stay were the three major end points for outcomes analysis.

**Results:**

100 % of the patients received antibiotics therapy before and after operation. 45 patients received Fluoroquinolones (48.4 %), 41 patients received Cephalosporins (44.1 %) and 7 patients received Carbapenems (7.5 %). We found no statistical difference between antibiotics selection and the occurrence of advanced appendicitis (P = 0.3337). Both the monovariate analysis and multivariate analysis showed no statistical difference between antibiotics selection and the postoperative complications (P > 0.05). The average stay of patients receiving Fluoroquinolones was 2.6 days shorter than patients who received Cephalosporins (P = 0.0085).

**Conclusion:**

It is a lack of a standardized guideline for antibiotics selection in our hospital. All the antibiotics prescription were empirical. We tended to choose high levels of antibiotics, pay insufficient attention to the anaerobic bacteria and have a long duration of antibiotics therapy. We also found that antibiotics selection bore no relationship with the occurrence of advanced appendicitis and postoperative complications. Fluoroquinolones may lead to a shorter hospital stay, but this result may also be affected by the fewer underlying diseases and lower severity of the patients.

## Background

Acute abdomen pain is one of the most common presenting complaints to the emergency department, and appendicitis is one of the most common underlying etiologies of an acute abdomen. Because of its acute onset, culture guided antibiotic therapy is limited when treating a patient with appendicitis. Hence, the effectiveness of empirical antibiotics is crucial. Currently, there is no guideline or general recommendation for antibiotics use for appendicitis in China. Antibiotics selection is usually based on individual practitioner preference with no definitive basis. The specific aim of this study was to analyze the antibiotics use status for acute appendicitis undergoing emergency appendectomy at a large tertiary care hospital in China, and find out whether there is a relationship between antibiotics selection and outcomes of acute appendicitis.

## Results

### Age and gender

There were 95 appendectomies performed during the study period. Two of the 95 patients were allergic to Fluoroquinolones, leaving 93 cases for analysis in total. There were no children or pregnant women. The age varied from 19 to 88 years old, with an average of 40.3 and standard deviation of 17.3. Among all patients, 44/93 (47.3 %) were male and 49/93 (52.7 %) were female.

### Pathogen

No patient underwent peritoneal fluid culture or tissue culture, so no pathogen results were reported. All antibiotics selections were empirical.

### Antibiotics type

The usage of antibiotics categories was shown in Table 1. There were only three types of antibiotics used for the patients, Fluoroquinolones (Levofloxacin, Moxifloxacin), Cephalosporins (Cefuroxim, Cefotaxime, Ceftazidime, Cefatriaxone, Cefepime), and Carbapenems (Ertapenem, Meropenem, Imiperem). Cephalosporins were mostly administered with Metranidazole together.

**Table 1:** Categories of antibiotics use

Antibiotics	Cases	Percentage (%)
Fluoroquinolone	45	48.4
Cephalosporins	41	44.1
Cephalosporins mono-therapy	7	7.5
Cephalosporins and metranidazole	34	36.6
Carbapenems	7	7.5
Total	93	100

#### Underlying diseases of the patients

59 Patients had none or only 1 underlying disease, and the rest 34 patients had more than two kinds of underlying diseases. Patients in the Carbapenems group was very few,so we only analyzed the Fluoroquinolones and Cephalosporins group. No relationship was shown between the antibiotics selection and the underlying disease (P = 0.3180), which is illustrated in Table 2.

**Table 2:** Antibiotics selection and underlying disease status

Antibiotics	Fewer disease (%)	More diseases (%)	Total
Fluoroquinolone	31 (68.9)	14 (31.1)	45
Cephalosporins	24 (58.5)	17 (41.5)	41
Carbapenems	4 (57.1)	3 (42.9)	7
Total	59 (63.4)	34 (36.6)	93

### Duration of antibiotics prescription

From the time when a diagnosis of appendicitis was suspected until the patients were discharged home, all patients were treated with IV antibiotics before and after surgery. The average duration of antibiotics prescription was 5.7 days. The mean duration of the mild appendicitis group was 5.2 days while the advanced appendicitis group was 7.1 days.

### The first diagnosis doctor

In many emergency departments in China, specialty doctors such as those from general medicine and surgery are often the first doctors in charge of the patient after being triaged. The doctor at the first point of contact usually decides the antibiotics use. Among the 93 patients, 65 patients were first attended to by the general surgeons (69.9 %); whilst 28 patients first presented to the internal medicine physicians (30.1 %). The Carbapenems group was too small to analyze,so we only analyzed the Fluoroquinolones and Cephalosporins group. Statistical analysis showed no correlation between first diagnosis doctor and antibiotics selection (P = 0.6850), which is illustrated in Table 3.

**Table 3:** First diagnosis doctor and antibiotics selection

Antibiotics	Medicine (%)	Surgical (%)	Total
Fluoroquinolone	15 (33.3)	30 (66.7)	45
Cephalosporins	12 (29.3)	29 (70.7)	41
Carbapenems	1 (14.3)	6 (85.7)	7
Total	28 (30.1)	65 (69.9)	93

### Mild and advanced appendicitis

68 cases were mild appendicitis (73.1 %), 25 cases were advanced appendicitis (26.9 %). Antibiotics usage had no statistical significance to the severity of appendicitis (P = 0.3337), which is illustrated in Table 4. Among all the 93 appendicitis, only one case had simple appendicitis, the others had suppurative, gangrenous or perforated appendicitis.

**Table 4:** Appendicitis and antibiotics type

Antibiotics	Mild (%)	Advanced (%)	Total
Fluoroquinolones	36 (80.0)	9 (20.0)	45
Cephalosporins	27 (65.9)	14 (34.1)	41
Carbapenems	5 (71.4)	2 (28.6)	7
Total	68 (73.1)	25 (26.9)	93

### Hospital stay

All the 93 patients showed eventual improvement and were discharged from the hospital. The length of stay varied from 1 to 34 days with a mean of (5.7 ± 5.1), the median stay was 4 days. The oldest patient in the study an 88-year-old female had the longest hospital stay of 34 days. The mean stay of the Fluoroquinolones group was 4.2 days while the Cephalosporins group was 6.8 days. The patients receiving Fluoroquinolones had a shorter length of stay by up to 2.6 days versus those given Cephalosporins (P = 0.0085).

A separate analysis about the Cephalosporins group was done. The average stay of Cephalosporins mono-therapy group was 5.6 days, and the average stay of Cephalosporins plus Metranidazole group was 7.1 days. Statistical analysis shows the hospital stay has no association between the Cephalosporins mono-therapy group and the Cephalosporin plus Metranidazole group (P = 0.5330).

### Postoperative complications

There was no re-operation or re-admission in the 93 patients. 11 Patients had SSIs (11.8 %), including 5 in the Fluoroquinolones group, 5 in the Cephalosporins group, 1 in the Carbapenems group. The number of the Carbapenems group was too small to analysis. A monovariate analysis was performed to compare the other two groups and found no statistical difference between the antibiotics selection and the SSIs (P = 0.8755), which was illustrated in Table 5. Multivariate analysis was carried out, but also no relationship was found between the antibiotics selection and the SSIs (P = 0.9184), which was illustrated in Table 6.

**Table 5:** Antibiotics type and SSIs

Antibiotics	No. of non-SSIs (%)	No. of SSIs (%)
Fluoroquinolones	40 (88.9)	5 (11.1)
Cephalosporins	36 (87.8)	5 (12.2)
Carbapenems	6 (85.7)	1 (14.3)
Total	82 (88.2)	11 (11.8)

**Table 6:** Multivariate logistic regression analysis of SSIs

Factors	P	OR
Age	0.7821	1.540
Gender	0.1251	0.124
Underlying disease status	0.4862	2.404
First diagnosis doctor	0.9723	1.041
Length of hospital stay	0.9971	5.617
Antibiotics type	0.9184	0.001

## Discussion

In this study, all appendectomies were performed via an open incision over McBurney’s point due its lower cost—because the local health insurance coverage is limited to US $500 per appendectomy, and laparoscopic procedures generally cost upwards of US $1000.

### Antibiotics and the occurrence of advanced appendicitis, length of hospital stay and SSIs

In our study, we analyzed the relationship between choice of antibiotics and development of advanced appendicitis, and failed to find positive correlation. It was indicated that higher level antibiotics prophylaxis usage failed to prevent the development of advanced appendicitis. The average hospitalization time in our hospital was 5.7 days. This result is comparable to other countries (Ohtani et al. 2012; Beek et al. 2015). We found Fluoroquinolones provided an average of 2.6 days shorter of hospital stay compared to Cephalosporins. However, this result may be complicated by patient’s baseline condition. The shorter hospital stay in Fluoroquinolones group may be the results of fewer underlying diseases and lower severity of the cases (see Tables 2, 4). In addition, Jeon et al. even suggested Fluoroquinolones should not to be the first choice of patients with appendicitis (Jeon et al. 2014). Hence, we cannot conclude Fluoroquinolones are superior to Cephalosporins. Prevention of postoperative complications, especially SSIs is a major purpose of the use of antibiotics. In our study, we found no difference between the antibiotics selection and the SSIs. This is similar to other studies. A prospective surveillance in Malaysia shows no significant association between choices of antibiotics with SSI (P = 0.299) (Oh et al. 2014). In our study, 11.8 % patients had SSIs, while the incidence of SSIs in appendectomy was about 5.56 % in Korea (Park et al. 2015). The higher rate of SSIs in our report may be due to the open appendectomy. As compared with open appendectomy, laparoscopic appendectomy is associated with a 50 % reduction of skin infection (Flum 2015).

### Antibiotics selection and anti-anaerobe therapy

Appendicitis infection is a mix of both aerobes and anaerobes. According to the treatment guidelines (Bratzler and Hunt 2006; Sartelli et al. 2013), appendicitis antibiotics therapy must cover gram negative rods and anaerobes. For mild appendicitis, the suggestion is a single dose of Cefoxitin, Ampicillin/Sulbactam, the combination of Cefazolin plus Metronidazole, or, Clindamycin plus one of the following: Ciprofloxacin, Levofloxacin, Gentamicin, or Aztreonam. For advanced appendititis, the guidelines suggest either monotherapy with a beta-lactam/beta-lactamase inhibitor (Piperacillin-Tazobactam or Ticarcillin-Clavulanate) or the combination of a third-generation Cephalosporin plus Metronidazole. Alternative choices are Fluoroquinolones plus Metronidazole or monotherapy with a Carbapenem.

According to our statistics, our hospital tends to choose higher level antibiotics such as moxifloxacin, third/fourth generation of Cephalosporins and Carbapenems, and use mono-therapy of Cephalosporins or Fluoroquinolones without Metronidazole compared to the international guideline. In our study, we found only 41/93 (44.1 %) patients had anti-anaerobe therapy (Metronidazole or Carbapenems). In addition, Cephalosporins plus Metronidazole failed to show statistical significance compared to Cephalosporins mono-therapy. These two results are contrary to the high anaerobe infection rate found in the literature (Zhuchenko 2016). We proposed the following reasons to explain this contradiction: (1) anaerobes are commensal enteric bacteria. The high rate of anaerobe detection in appendicitis may be due to contamination. The role of anaerobic bacteria in appendicitis was uncertain (Guinane et al. 2013; Zhuchenko 2016). (2) Patient may benefit solely from surgical appendectomy regardless of post-operative antibiotic therapy. Even so, this does not mean we can choose any antibiotics at our will. We should take many things into consideration when selecting antibiotics, such as the previous studies, the international guidelines, the bacterial culture results, the severity of appendicitis and the basic situation of the patient.

#### Duration of antibiotics prescription

In our study, all patients were treated with antibiotics before and after surgery. The average duration of the mild appendicitis group was 5.2 days while the advanced appendicitis group was 7.1 days. This was a long period, especially for the mild appendicitis. The international guidelines suggested a single dose of antibiotics for mild appendicitis, and a 5–7 days antibiotics prescription for advanced appendititis (Bratzler and Hunt 2006; Sartelli et al. 2013; Solomkin et al. 2010). Some studies even pointed out that 3 days of antibiotics treatment was equally effective as 5 days (van Rossem et al. 2014; Reibetanz and Germer 2014). The current situation of long antibiotic treatment in our hospital should attract our attentions.

## Conclusion

In our study, we found that antibiotics selection has no relationship with the occurrence of advanced appendicitis and SSIs. Notably Fluoroquinolones may lead to a shorter hospital stay, but this result may also be affected by the fewer underlying diseases and lower severity of the cases. Besides, we also found that the antibiotics choice for appendicitis was usually based on the personal experience. We had an overutilization of higher level antibiotics, and paid insufficient attention to the treatment of anaerobic bacteria. In addition, the duration of antibiotics therapy was longer than the suggestion of international guidelines. All the above showed a lack of standardized guideline for antibiotics usage in China.

## Methods

Peking Union Medical College Hospital (PUMCH) is a large tertiary care hospital in China with about 1800 beds, and covering the medical service in North China. The case records were reviewed for all patients who underwent emergency appendectomy at PUMCH between June 1, 2011 to May 30, 2012, excluding age <18, pregnant women, and patients allergic to the commonly use antibiotics. Data retrieved included demographic data, gender, age, underlying disease, antibiotic type, the duration of antibiotics prescription, the specialty doctor who was the first diagnosis doctor, type/severity of appendicitis, postoperative complications, length of hospital stay, and final clinical outcome.

Nine kinds of diseases have been taken into consideration as the underlying disease. They were cerebrovascular disease, coronary heart disease, hypertension, diabetes, chronic cardiac insufficiency, chronic pulmonary disease, chronic kidney insufficiency, chronic hepatic insufficiency and tumor. The patients with none or one underlying disease were classified into fewer disease group, and patients with two or more underlying diseases were classified into more diseases group, The patients were categorized according to the appearance of the peritoneal cavity, the appearance of appendix at the time of operation, and the histo-pathological results. All the patients were divided into two groups—mild group and advanced. We defined simple appendicitis and suppurative appendicitis as mild appendicitis, whilst gangrenous appendicitis and perforated appendicitis were classified as advanced appendicitis. Postoperative complications included surgical site infections (SSIs) such as wound infection, secondary peritonitis, intra-abdominal abscess and other intra-abdominal infection within 30 days after the appendectomy, re-operation and re-admission. In our study, the occurrence of advanced appendicitis, postoperative complications and the length of hospital stay were recorded as the three main outcomes for analysis.

SPSS17.0 was used to analysis these data. Continuous variables expressed as mean ± SD. Continuous variables were compared with *t*-tests. Univariate associations were explored with Pearson’s χ^2^ tests for independent proportions. Logistic regression was used to carry out the multivariate analysis. P < 0.05 was used to indicate statistically significant differences.
